# Complex Probiotics Suppress Inflammation by Regulating Intestinal Metabolites in Kittens

**DOI:** 10.3390/ani15020272

**Published:** 2025-01-19

**Authors:** Shimin Zhu, Musu Zha, Yanan Xia

**Affiliations:** 1 Key Laboratory of Dairy Biotechnology and Engineering, Ministry of Education, Inner Mongolia Agricultural University, Hohhot 010018, China; 13603576436@163.com; 2 Inner Mongolia Key Laboratory of Dairy Biotechnology and Engineering, Inner Mongolia Agricultural University, Hohhot 010018, China

**Keywords:** probiotics, kitten, metabolomics, metabolites

## Abstract

Cats are popular companions for humans, and their health is of importance to a growing number of pet owners. The juvenile period is a critical stage of cat growth; in particular, the metabolic activity of the intestinal microbiome of kittens is critical for processing nutrients and supporting overall health. In this study, the effect of complex probiotics on the intestinal health of kittens was explored through a metabolomics analysis. Twenty-four healthy kittens were randomly assigned to two groups (*n* = 12): the control group was provided a basal diet and the probiotics group was provided the basal diet supplemented with complex probiotics (given at the same time daily). The kittens were acclimatized for 5 days, and the experiment was conducted for 14 days. We collected feces from each kitten on days 1 and 14 for metabolomic analyses. Compared to the control, the probiotics group had significantly higher (*p* < 0.05) methylmalonylcarnitine, lysyl-hydroxyproline, phenylpropionylglycine, and vitamin K3 levels, and significantly lower (*p* < 0.05) gamma-glutamyl-L-putrescine, cis-gondoic acid, myristic acid, 12,13-DiHOME, and glycodeoxycholic acid levels. The results of this study suggest that complex probiotics promote intestinal health in kittens by regulating changes in various metabolites in the intestine and may have a mitigating effect on intestinal inflammation.

## Simple Summary

The juvenile period is a critical stage of cat growth; in particular, the metabolic activity of the kitten’s intestinal microbiota is critical to overall health. This study investigated the effects of a probiotic complex on the intestinal health of kittens. We found that complex probiotics significantly altered the levels of metabolites associated with inflammation and constipation in the intestines of kittens. This suggests that complex probiotics may promote intestinal health in kittens by modulating changes in various metabolites. This study provides a rationale for the development of probiotic supplements for kittens.

## 1. Introduction

The gastrointestinal (GI) tract plays a key role in animal health [1,2]. The intestinal microbiota is a complex microbial community within the GI tract that performs functions related to barrier protection, nutrition, metabolism, and immunity. Thus, the composition and status of the intestinal microbiota are closely related to the host’s health [3]. An imbalance in the intestinal microbiota can cause various problems in the host organism, resulting in diseases such as ulcerative colitis, Crohn’s disease, and colorectal cancer [4,5]. In the neonatal intestine, the establishment of the intestinal microbiota is thought to influence the development of the immune system after birth and long-term physical health [6].

Similarly, the intestinal health of cats is critical to their overall health, impacting nutrient absorption, immune function, mental health, and other functions. For example, one study reported significant differences in the intestinal microbiota between healthy cats and cats with inflammatory bowel disease (IBD), suggesting a potential link between intestinal microbiota composition and disease development [1]. Kittens are particularly susceptible to intestinal microbiota disorders because of their underdeveloped intestinal tract and lack of stable intestinal microbiota. As a result, kittens are more likely to develop intestinal health problems and exhibit symptoms such as vomiting, diarrhea, reflux, and weight loss [7]. Weaning is a common stressor in kittens and is associated with an increase in infection risk and GI discomfort [6,8,9,10].

Probiotics are beneficial microorganisms that have a variety of positive effects; through exerting changes in intestinal metabolites and intestinal microbiota, probiotics can inhibit pathogenic bacteria colonization, maintain intestinal flora homeostasis, and promote nutrient digestion and absorption [1,10]. Thus, probiotics are increasingly used to treat GI diseases. In pets, several studies have demonstrated the positive effects of probiotics in the treatment of IBD, relief of acute gastroenteritis, and prevention of allergies; probiotics may even have the potential to replace antibiotics in animal production [3,11,12].

A number of studies have examined the effects of probiotics on the intestinal microbiota of cats. For example, *Enterococcus faecium* SF68 was shown to maintain fecal microbial diversity [13,14]; *Bacillus licheniformis* fermentation products improved the fecal microbiota [5]; and *Lactobacillus plantarum* L11 improves intestinal health and immune function in cats while also being implicated in lipid metabolic pathways [11]. Moreover, multi-strain complex probiotics can promote intestinal health in cats by modulating the intestinal flora, and probiotic and enzyme complexes can reduce the proportion of harmful bacteria in the cat intestine and improve the intestinal flora structure [15].

In kittens, the metabolic activity of the intestinal microbiome is critical for nutritional processing and overall health; however, few studies have investigated the effects of probiotics on feline intestinal metabolites, and the mechanism of action of probiotics on intestinal health in kittens remains unclear. Therefore, the aim of this study was to elucidate how probiotics affect the intestinal health of kittens by analyzing the metabolites produced through microbial activity using a metabolomics approach.

## 2. Materials and Methods

### 2.1. Animals and Experimental Design

**Figure 1:**
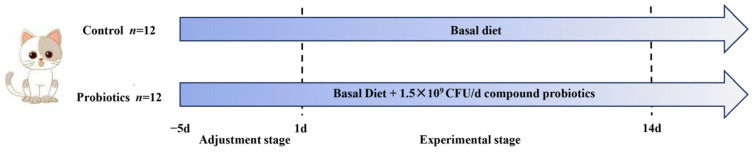
Schematic diagram of the experimental design. Kittens in the control group were fed a basal diet. Kittens in the probiotics group were fed a basal diet plus 1.5 × 10^9^ CFU/day complex probiotics.

Figure 1 presents an overview of the experimental design. Twenty-four healthy kittens aged 3–4 months were randomly divided into control and probiotics groups (*n* = 12 each) based on their weight and sex. Initially, there was no significant difference between the two groups of kittens in terms of mean baseline body weight (*p* > 0.05). The kittens were acclimatized for 5 days, and then the experimental period lasted for 14 days. During this time, all kittens were fed a basal diet; the kittens in the probiotics group were additionally fed a complex probiotics preparation (*Bifidobacterium animalis* subsp. *lactis* BX-259, *Lactiplantibacillus plantarum* LP-301, *Lacticaseibacillus rhamnosus* LR-78 in a 1:1:1 ratio; 1.5 × 10^9^ CFU/day/kitten, Inner Mongolia Agricultural University, Hohhot, China) at the same time every morning. We collected fresh feces from each kitten on the mornings of experimental days 1 and 14. From the center of the fecal mass, we sampled 2 g of fresh feces. The 2 g sample was divided into two 2 mL sterile Eppendorf (EP) tubes and stored immediately at −80 °C for metabolomic analyses.

All kittens were immunized and dewormed as needed before inclusion in the experiment. No kittens received any medication (e.g., antibiotics) that could cause changes in intestinal microbiota within 1 month before the start of the experiment. The kittens were housed individually in cages (50 cm × 32 cm × 38 cm). Each cage contained a litter tray and food bowl. Each kitten had its own assortment of toys and scratching poles to enrich the environment and interacted with humans. All cages were cleaned and sterilized regularly. The kittens were provided with an ample daily supply of clean drinking water and commercial dry food (C3 PEPTIDO milk cake cat food; Partido (Linyi) International Biotechnology Co., Ltd., Linyi, Shandong, China. The specific ingredient list is shown in Table 1).

This study was conducted in accordance with the guidelines for the use and care of laboratory animals and was approved by the Laboratory Animal Welfare and Ethics Committee of Inner Mongolia Agricultural University (no. NND2023098). Euthanasia was not the endpoint of the study; all kittens were subsequently adopted into private homes.

### 2.2. Fecal Targeted Metabolomics

From each kitten, a 0.1 g fecal sample was weighed in a 2 mL homogenizing tube containing 1.0 mm zirconia beads. After 2 mL of 50% methanol (Merck, Darmstadt, Germany) water was added to each tube, the mixture was homogenized twice for 30 s at 3 m/s. We centrifuged the tubes (12,000× *g*, 10 min) and transferred 40 μL of the resulting supernatants into new sterile EP tubes. After 20 μL of 200 mM 3-nitrophenylhydrazine and 20 μL of 120 mM EDC ((N-(3-dimethylaminopropyl)-N′-ethyl carbodiimide hydrochloride)-6% pyridine) were added to the supernatant, the EP tubes were mixed via shock centrifugation and then incubated in a water bath at 40 °C for 30 min. Next, we added 1920 μL of 50% methanol water to the tubes, which were shaken and centrifuged again. The resulting supernatants were filtered through a 0.22 µm microporous membrane filter for a targeted metabolomic analysis.

We used a liquid chromatography–mass spectrometry (LC-MS) platform (AB Sciex, Framingham, MA, USA) for targeted metabolomic determination of the fecal samples. We used an ultra-high-performance-liquid chromatography–quadrupole time-of-flight (UPLC-Q-TOF) system that was optimized according to the protocol of Li et al. [16]. The instrument requirements included mobile phases of 5% methanol in water +0.1% formic acid and 0.1% formic acid in methanol, with a flow rate of 0.4 mL/min, 1 μL per injection, and a column temperature of 40 °C.

### 2.3. Fecal Untargeted Metabolomics

From each kitten, a 0.05 g fecal sample was weighed in a 2 mL homogenizing tube containing 1.0 mm zirconia beads. After 500 μL of 40% methanol acetonitrile (Merck, Darmstadt, Germany) water was added, the mixture was homogenized twice for 30 s at 3 m/s. We centrifuged the tubes (12,000× *g*, 10 min) and transferred the resulting supernatants to sterile EP tubes, which were then evaporated until dry. The EP tubes were reconstituted with 50 μL of 50% acetonitrile in water and then centrifuged again. The resulting supernatants were filtered through a 0.22 µm microporous membrane filter for untargeted metabolomic analysis.

We used an LC-MS platform for the untargeted metabolomic determination of the fecal samples. We also used a UPLC-Q-TOF system optimized according to the protocol of Li et al. [16]. The instrumental requirements included a mobile phase of water and pure acetonitrile, a flow rate of 0.3 mL/min, 1 μL per injection, and a column temperature of 40°C. We used Progenesis Ql (Waters, Milford, MA, USA) for peak extraction, peak alignment, deconvolution, and normalization of the downstream data, followed by multivariate data analyses and metabolite annotations based on the Kyoto Encyclopedia of Genes and Genomes (KEGG) database to screen for differential metabolic pathways.

### 2.4. Statistical Analysis

Differences in metabolite contents between the control and probiotics groups were analyzed using the Wilcoxon rank-sum test. All experimental data were expressed as means ± standard error of the mean (SEM). We used R v4.3.2 (R Core Team, Vienna, Austria) for all statistical analyses. Significant differences were evaluated at a threshold of *p* < 0.05. We used the MetaboAnalyst 6.0 analysis platform (https://www.metaboanalyst.ca/faces/home.xhtml, accessed on 15 September 2024) to process data for the untargeted metabolomic analysis. Graphs were produced using the MetaboAnalyst 6.0 analysis platform, the R software package (R version 4.3.2), and OmicStudio (https://www.omicstudio.cn/home, accessed on 15 October 2024).

## 3. Results

### 3.1. Effects of Complex Probiotics in Fecal Targeted Metabolomics

**Figure 2:**
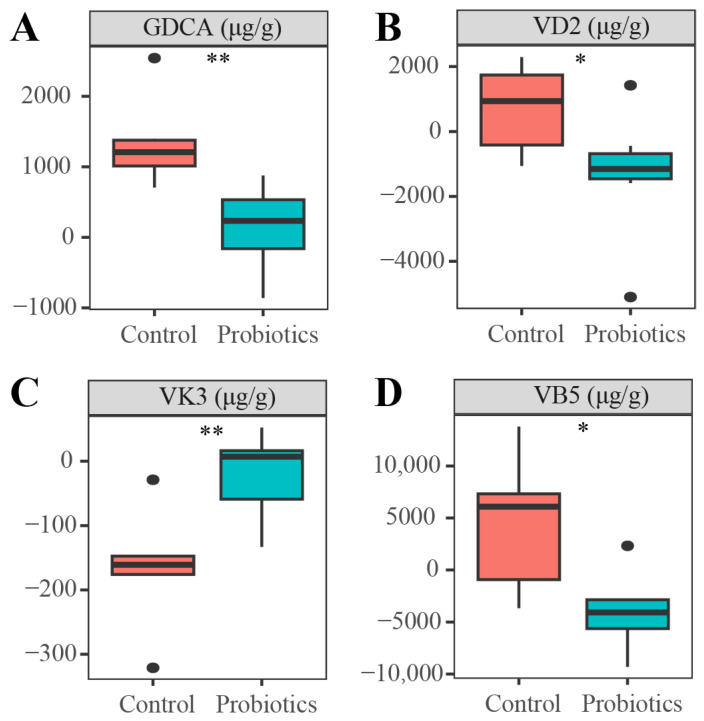
Differential metabolites identified in the targeted metabolomic analysis of kitten feces in the probiotics and control groups (*n* = 12 each): (**A**) Glycodeoxycholic acid (GDCA), (**B**) vitamin D2 (VD2), (**C**) vitamin K3 (VK3), and (**D**) vitamin B5 (VB5). Data are expressed as means ± standard error of the mean (SEM). Asterisks indicate significant differences (* *p* < 0.05, ** *p* < 0.01).

In the targeted metabolomic analysis of kitten feces, we examined 24 metabolites, including three bile acids, six vitamins, four short-chain fatty acids, two organic acids, and eight amino acids. Of these, four metabolites differed significantly between the probiotics and control groups (Figure 2): vitamin K3 (VK3), glycodeoxycholic acid (GDCA), vitamin D2 (VD2), and vitamin B5 (VB5). The VK3 levels were significantly higher, and the GDCA, VD2, and VB5 levels were significantly lower, in the probiotics group than in the control group (*p* < 0.05).

### 3.2. Effects of Complex Probiotics in Fecal Untargeted Metabolomics

**Figure 3:**
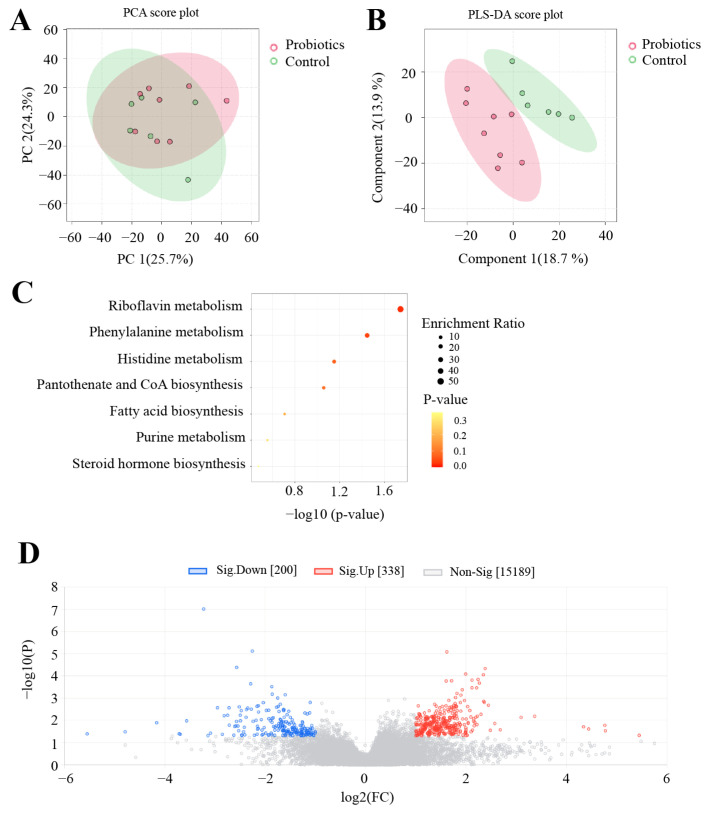
Effects of complex probiotics on the intestinal tract of kittens based on an untargeted metabolomics analysis (*n* = 12). (**A**) A plot of the principal component analysis (PCA) scores. (**B**) A lot of the partial least squares discriminant analysis (PLS-DA) scores. (**C**) A Kyoto Encyclopedia of Genes and Genomes (KEGG) enrichment analysis, where terms with −log10(*P*) > 1.3 were considered significantly enriched. Circle size represents the degree of metabolite enrichment in the pathway. (**D**) Volcano plot of metabolites; red and blue dots represent up- and downregulated metabolites, respectively.

To further examine the effects of the complex probiotics preparation on the intestinal tracts of kittens, we performed untargeted metabolomic analyses of kitten feces. First, we assessed metabolites between the control and probiotics groups via principal component analysis (PCA) and partial least squares discriminant analysis (PLS-DA) score plots. PCA is an unsupervised pattern recognition analytical method that visualizes differences among samples in a multidimensional space. PLS-DA achieves a maximum separation of samples by mathematically modeling different groups. The PCA plot showed non-significant separation between the two groups (Figure 3A). The PLS-DA score plot indicated differences between the two groups (Figure 3B).

**Figure 4:**
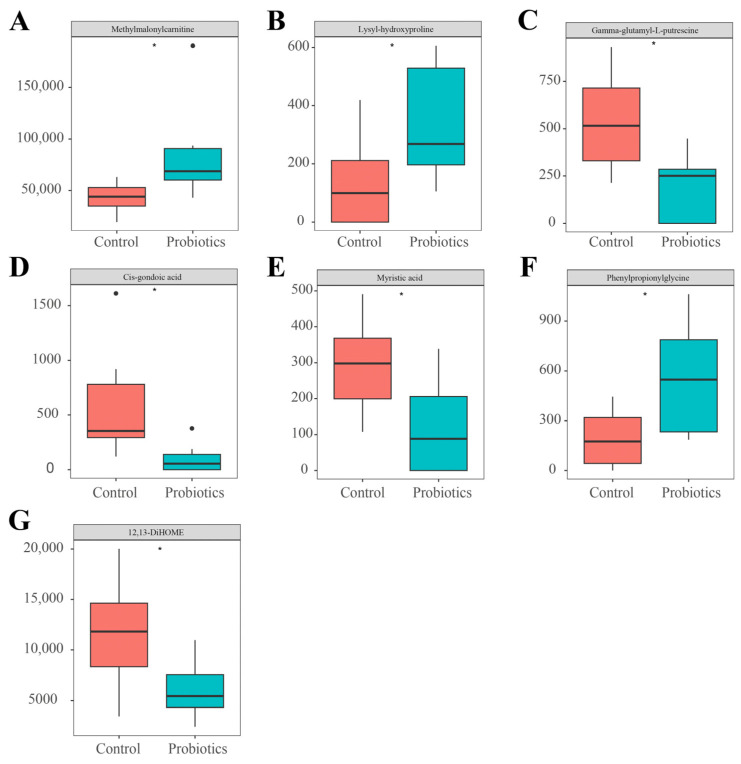
Differential metabolites in the feces of kittens in the control and probiotics groups on experimental day 14 based on an untargeted metabolomic analysis (*n* = 12). (**A**) Methylmalonylcarnitine. (**B**) Lysyl-hydroxyproline. (**C**) Gamma-glutamyl-L-putrescine. (**D**) Cis-gondoic acid. (**E**) Myristic acid. (**F**) Phenylpropionylglycine. (**G**) 12,13-DiHOME. Data are expressed as means ± SEM. Asterisks indicate significant differences (* *p* < 0.05).

In the untargeted metabolomic analysis, we detected a total of 15,727 metabolites. Based on the screening conditions of variable importance in projection > 1, |log2(fold change)| > 1, and *p* < 0.05, we screened a total of 538 significantly changed differential metabolites. Compared with the control group, 338 metabolites were upregulated and 200 metabolites were downregulated in the probiotics group (Figure 3D). We matched 44 differential metabolites (Table 2), first against an internal standard library and then against the online Human Metabolome Database (https://hmdb.ca/, accessed on 25 September 2024) and the LIPID MAPS database (https://www.lipidmaps.org/, accessed on 25 September 2024). We found significantly higher methylmalonylcarnitine, lysyl-hydroxyproline, and phenylpropionylglycine levels and significantly lower gamma-glutamyl-L-putrescine, cis-gondoic acid, myristic acid, and 12,13-DiHOME levels in the probiotics group compared to the control group (*p* < 0.05). Figure 4 shows differences in these metabolites between the two groups. Next, we performed a KEGG enrichment analysis of the differential metabolites using the MetaboAnalyst 6.0 analysis platform. Probiotics had significant effects on two metabolic pathways: riboflavin metabolism and phenylalanine metabolism (*p* < 0.05; Figure 3C).

### 3.3. Correlation Analysis

**Figure 5:**
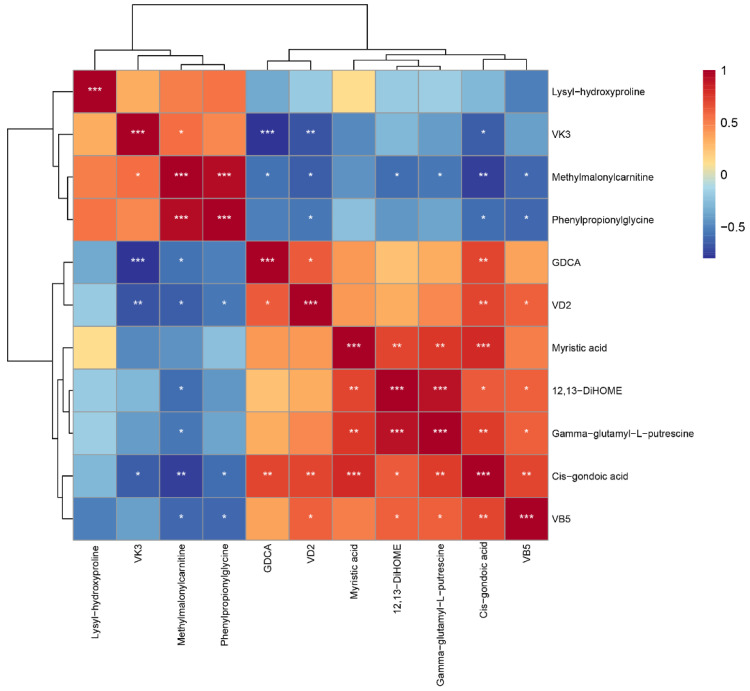
Spearman correlations between differential metabolites in the metabolomic analysis of the feces of kittens in the control and probiotics groups. Red and blue indicate positive and negative correlations, respectively. Darker colors indicate stronger correlations. Data are expressed as means ± SEM. Asterisks indicate significant differences (* *p* < 0.05, ** *p* < 0.01, *** *p* < 0.001).

Figure 5 presents the Spearman correlation analysis results. Phenylpropionylglycine was significantly negatively correlated with cis-gondoic acid (R = −0.59, *p* < 0.05) and significantly positively correlated with methylmalonylcarnitine (R = 0.93, *p* < 0.001). Notably, 12,13-DiHOME showed significant positive correlations with cis-gondoic acid (R = 0.63, *p* < 0.05), gamma-glutamyl-L-putrescine (R = 0.92, *p* < 0.001), and myristic acid (R = 0.69, *p* < 0.01) and a significant negative correlation with methylmalonylcarnitine (R = −0.58, *p* < 0.05). VB5 showed significant positive correlations with cis-gondoic acid (R = 0.69, *p* < 0.01), gamma-glutamyl-L-putrescine (R = 0.62, *p* < 0.05), and 12,13-DiHOME (R = 0.61, *p* < 0.05) and significant negative correlations with phenylpropionylglycine (R = −0.61, *p* < 0.05) and methylmalonylcarnitine (R = −0.62, *p* < 0.05).

## 4. Discussion

The intestinal microbiota of kittens is underdeveloped and lacks stability, rendering kittens susceptible to intestinal microbiota disorders and various health problems [6,7,8,9,10]. Research on probiotics has identified various positive effects in pet animals, such as treating IBD and relieving acute gastroenteritis. Cats have been the subject of a number of studies on probiotics; however, such studies have mainly focused on adult cats, with relatively few being conducted on kittens. Therefore, the aim of this study was to investigate the effects of complex probiotic supplementations on the intestinal health of kittens.

Studying feces using metabolomics can help to elucidate the biochemical processes of the host intestine. Therefore, we analyzed the effects of complex probiotics on the intestinal tract of young kittens through fecal metabolomics analyses. In the present study, we found that the levels of methylmalonylcarnitine and lysyl-hydroxyproline were significantly higher in the probiotics group than in the control group. Methylmalonylcarnitine is an organic fatty acid metabolite that is associated with the fatty acid β-oxidation rate [17]; acetyl-CoA and succinyl-CoA produced via β-oxidation are gluconeogenic substrates that provide energy to prevent hypoglycemic episodes and have the potential to alleviate fatigue in the host. Dogs with IBD were reported to have significantly reduced methylmalonylcarnitine levels in their feces [18]. Lysyl-hydroxyproline is an organic compound containing two α-amino acids linked by a peptide bond. A previous study reported elevated intestinal lysyl-hydroxyproline levels after oral administration of *Lactobacillus plantarum* 12 to mice suffering from colon cancer [19].

By querying the metabolites associated with intestinal inflammation, we noted relevant metabolites with significantly lower levels in the probiotics group: gamma-glutamyl-L-putrescine, cis-gondoic acid, and myristic acid. Significantly higher levels of gamma-glutamyl-L-putrescine were observed in the feces of mice suffering from chronic ulcerative colitis [20]. Cis-gondoic acid (systematic name: 11Z-eicosenoic acid) is an organic compound with an aliphatic tail. A previous study reported that pigs suffering from chronic intestinal inflammatory diseases had significantly lower cis-gondoic acid levels in their feces after treatment [21]. Cis-gondoic acid was also detected in the feces of both IBD and healthy dogs [22]. Myristic acid is an organic compound with an aliphatic tail which is positively correlated with the pro-inflammatory cytokine interleukin (IL)-6 [23]. Increased myristic acid levels have been detected in cats with chronic intestinal disease [24]. There was a significant positive correlation between cis-gondoic acid and myristic acid (Figure 5). We hypothesize that complex probiotics can promote intestinal health and potentially reduce intestinal inflammation by modulating inflammation-related metabolites in the intestine of kittens.

In addition, we have identified several metabolites associated with the physical health of kittens, including phenylpropionylglycine and 12,13-DiHOME. Phenylpropionylglycine is an organic compound containing α-amino acids. We found that phenylpropionylglycine levels were significantly higher in the probiotics group than in the control group, and this difference may be due to increased levels of methylmalonylcarnitine. Because phenylpropionylglycine is an important metabolite of fatty acid β-oxidation, its concentration is significantly negatively correlated with fatty acid levels in the liver [25]. Phenylpropionylglycine was previously found to be more abundant in the feces of healthy cats than in the feces of cats with chronic kidney disease [26]. Phenylpropionylglycine produces anti-adipogenic effects by attenuating the adipocyte differentiation of 3T3-L1 cells through the inhibition of the adiponectin PPAR pathway [27]. Meanwhile, 12,13-DiHOME (systematic name: 12,13-dihydroxy-9Z-octadecenoic acid) is a long-chain fatty acid with an aliphatic tail. We found that 12,13-DiHOME levels were significantly lower in the probiotics group than in the control group. Another study found that 12,13-DiHOME altered the expression of PPARγ-regulated genes in human dendritic cells while also reducing the secretion of the anti-inflammatory cytokine IL-10 and the number of Treg cells in the intestines of neonates at high risk of allergy or asthma [28]. There was a significant positive correlation between 12,13-DiHOME and cis-gondoic acid (Figure 5). We speculate that 12,13-DiHOME has a promoting role in allergic inflammation. Overall, these results suggest that supplementation with complex probiotics may positively impact kitten health by reducing metabolites associated with inflammation or other organ health.

Through targeted metabolomics analyses, we identified four metabolites exhibiting significant differences: VK3, GDCA, VD2, and VB5. Among them, the content of VK3 was significantly higher in the probiotics group. An in vitro study showed that VK3 inhibits inflammation [29]. Additionally, VK may protect against dextran sodium sulfate-induced colitis and ameliorate intestinal damage in mice by inhibiting the production of inflammatory cytokines [30]. Another study reported that VK3 induced apoptosis in tumor cells, thus exerting anti-tumor effects [31]. Laying hens improved their immune function and intestinal antioxidant capacity after consuming diets supplemented with moderate amounts of vitamin A (7000 IU/kg) and VK3 (2.0 mg/kg) [32]. Levels of three other metabolites, GDCA, VD2 and VB5, were significantly lower in the probiotics group. A study reported significantly higher levels of GDCA in human diarrhea [33]. The results of our targeted metabolomics analyses were consistent with the results of the untargeted metabolomics analyses; therefore, we hypothesized that the complex probiotics could improve intestinal health in kittens by modulating inflammation-related metabolites in the intestine. Notably, we found significantly lower VB5 levels in the probiotics group than in the control group. VB5 is a water-soluble vitamin also known as pantothenic acid. A previous study reported that VB5 improved the health status of weaned piglets [34]; however, another found significantly lower levels of VB5 in mice fed a high-fat diet [35]. Through a search tool for interactions of chemicals (STITCH) analysis, we found that phenylpropionylglycine interacted directly with hexanoyl glycine, which is associated with fatty acid metabolism and is lower in obese individuals [36,37]. Thus, probiotic complex supplementations likely promoted the fat absorption capacity of the intestinal tract of the kittens. The commercial dry food we fed the kittens used in this study contained only about 18% fat and was not consideredto be high-fat food. Therefore, we conclude that the reduced VB5 levels in the probiotics-supplemented kittens were caused by the complex probiotics.

**Figure 6:**
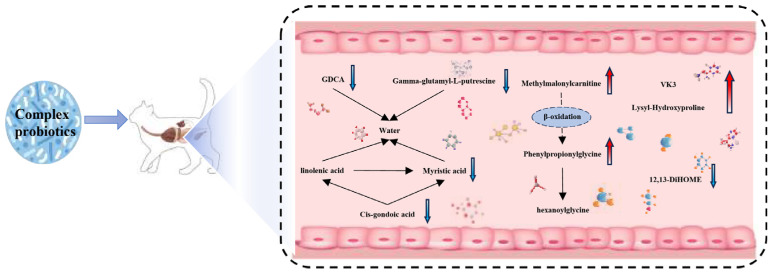
Effect of complex probiotics on intestinal metabolites in kittens.

Figure 6 summarizes the mechanism of action of the complex probiotics in the intestinal tract of kittens; metabolite interactions were determined via STITCH analysis. GDCA, gamma-glutamyl-L-putrescine, myristic acid, and cis-gondoic acid directly or indirectly interact with linolenic acid and water. Linolenic acid has been reported to improve the intestinal barrier and alleviate inflammation in mice [38,39], which suggests that complex probiotics may have a mitigating effect on intestinal inflammation in kittens. These metabolites were also associated with intestinal water volume, suggesting that complex probiotics may promote intestinal health in kittens by improving fecal scores. By correlation analysis we found that GDCA was significantly positively correlated with cis-gondoic acid. Cis-gondoic acid was positively correlated with myristic acid and gamma-glutamyl-L-putrescine. This indicates the credibility of the interactions we found through STITCH analysis. These results suggest that the complex probiotics improved intestinal health in kittens by modulating their intestinal metabolites and may potentially have a mitigating effect on intestinal inflammation in kittens.

## 5. Conclusions

The present findings suggest that supplementation with complex probiotics can have a significant effect on the intestinal health of kittens. Complex probiotics significantly altered metabolite levels in the intestinal tracts of kittens, including myristic acid, 12,13-DiHOME, and VK3, which positively affected pro- and anti-inflammatory cytokines. Therefore, further studies could be conducted to determine the role of complex probiotics in alleviating intestinal inflammation. Through STITCH analysis, we also found that several of the metabolites altered by complex probiotics supplementation (e.g., gamma-glutamyl-L-putrescine and GDCA) may contribute to ameliorating constipation. Overall, the present findings highlight the potential of complex probiotics for use as nutritional supplements in kittens and provide a rationale for the development of probiotic supplements for kittens.

## Figures and Tables

**Table 1:** Composition and nutrient content of experimental diets ^1^.

Items	Content (%)
Ingredients	
Beef	20.00
Salmon	16.00
Mackerel	8.00
Purple potatoes	7.00
Chicken	7.00
Duck	5.00
Chickpea	4.00
Chicken oil	3.00
Other substances ^2^	30.00
Nutrient content	
Crude protein	35.00
Crude fat	18.00
Moisture	10.00
Ash	8.00
Crude fiber	3.50
Calcium	1.30
Phosphorus	1.20
Taurine	0.35
Omega-6	2.60
Water-soluble chloride	0.30

^1^ Sourced from labeling instructions and related information provided by the manufacturer. ^2^ Other substances include deep-sea salmon fish oil, fresh whole eggs, olive oil, butter, linseed oil, cheese, dairy colostrum, sheep milk powder, sugar beet pulp, dietary fiber, beer yeast powder, carrots, broccoli, pumpkin, pea, tomato, apple, pear, low fructose FOS, low poly galactose GOS, semen plantaginis, cranberry, yucca, chicory roof, rosemary, seaweed, and chlorella powder.

**Table 2:** Differential metabolites between the probiotics and control groups matched to databases based on the screening conditions of variable importance in projection > 1, |log2(fold change)| > 1, and *p* < 0.05.

Number	Metabolite Identity	Molecular Fomula	Mass-to-Charge Ratio (*m*/*z*)	Fold Change	*p*-Value	VIP	Retention Time
1	Cis-4-Decenoic acid	C_10_H_18_O_2_	170.2533	0.42	0.04	1.64	9.93
2	Menadione	C_11_H_8_O_2_	172.0854	0.43	0.02	1.39	9.82
3	Alprazolam	C_17_H_13_ClN_4_	308.5893	0.42	0.03	1.94	9.71
4	Progesterone	C_21_H_30_O_2_	314.2298	0.18	0.04	1.75	8.33
5	Monoethyl phthalate	C_10_H_10_O_4_	194.1222	0.32	0.03	2.03	8.12
6	L-Homocysteic acid	C_4_H_9_NO_5_S	169.0487	0.33	0.02	1.89	7.87
7	Dopamine 3-O-sulfate	C_8_H_11_NO_5_S	219.0321	0.47	0.04	1.83	7.87
8	Norrubrofusarin 6-beta-gentiobioside	C_26_H_30_O_15_	582.1328	0.32	0.04	1.49	7.84
9	Phenylacetic acid	C_8_H_8_O_2_	136.0447	0.34	0.03	1.97	7.84
10	6-(2-Carboxyethyl)-7-hydroxy-2,2-dimethyl-4-chromanone glucoside	C_20_H_26_O_10_	426.1406	0.32	0.02	2.23	7.83
11	4-Hydroxy-1H-indole-3-acetonitrile	C_10_H_8_N_2_O	156.1251	0.32	0.02	2.33	7.70
12	4-Aminohippuric acid	C_9_H_10_N_2_O_3_	194.0179	0.28	0.01	2.45	7.48
13	Pantothenic acid	C_9_H_17_NO_5_	205.1075	0.23	0.01	2.47	7.08
14	Glutarylcarnitine	C_12_H_21_NO_6_	261.1292	0.21	0.01	2.66	7.06
15	L-leucyl-L-proline	C_11_H_20_N_2_O_3_	228.1447	0.17	0.01	2.34	6.02
16	N-Undecanoylglycine	C_13_H_25_NO_3_	229.1986	0.37	0.02	1.99	5.70
17	Hydroxypropionylcarnitine	C_10_H_19_NO_5_	219.1186	0.43	0.02	1.99	5.66
18	2-Hydroxy-3-methylbutyric acid	C_5_H_10_O_3_	118.0553	0.20	0.01	2.67	5.17
19	Riboflavin	C_17_H_20_N_4_O_6_	376.1065	2.15	0.01	2.09	4.77
20	Methylmalonylcarnitine	C_11_H_19_NO_6_	247.1546	2.00	0.03	1.93	4.77
21	Gamma-glutamyl-L-putrescine	C_9_H_19_N_3_O_3_	217.1278	0.08	0.04	2.24	4.77
22	N2-Succinyl-L-ornithine	C_9_H_16_N_2_O_5_	232.0989	4.58	0.01	1.92	4.64
23	Lysyl-Hydroxyproline	C_11_H_21_N_3_O_4_	259.1462	2.13	0.03	1.80	4.13
24	Phenylpropionylglycine	C_11_H_13_NO_3_	193.0877	3.38	0.02	1.94	3.89
25	Aspartyl-Histidine	C_10_H_14_N_4_O_5_	270.0777	43.71	0.04	1.56	2.87
26	Guanine	C_5_H_5_N_5_O	135.141	3.60	0.04	1.72	2.19
27	N-gamma-L-Glutamyl-D-alanine	C_8_H_14_N_2_O_5_	218.0833	3.25	0.04	1.68	2.02
28	Catelaidic acid	C_22_H_42_O_2_	338.3171	0.18	0.01	2.24	17.01
29	Vaccenic acid	C_18_H_34_O_2_	282.2622	0.31	0.01	1.87	17.00
30	Beta-Elemonic acid	C_30_H_46_O_3_	454.7252	0.42	0.02	1.52	16.33
31	Cis-gondoic acid	C_20_H_38_O_2_	310.2857	0.18	0.01	2.06	16.16
32	Barogenin	C_27_H_42_O_4_	430.3404	0.37	0.02	2.05	14.68
33	5-Nonadecyl-1,3-benzenediol	C_25_H_44_O_2_	376.2962	0.41	0.04	1.67	14.49
34	4,8 dimethylnonanoyl carnitine	C_18_H_35_NO_4_	315.2392	0.38	0.03	2.26	13.86
35	3-Hydroxy-5, 8-tetradecadiencarnitine	C_21_H_37_NO_5_	369.2602	0.12	0.04	1.93	13.16
36	Trans-2-Dodecenoylcarnitine	C_19_H_35_NO_4_	327.2906	0.39	0.03	2.20	12.69
37	18-Oxocortisol	C_21_H_28_O_6_	376.1948	0.19	0.01	2.49	12.10
38	Myristic acid	C_14_H_28_O_2_	228.2067	0.40	0.02	2.32	11.70
39	12,13-DiHOME	C_18_H_34_O_4_	314.2391	0.43	0.04	2.20	11.65
40	Pelargonic acid	C_9_H_18_O_2_	158.2537	0.18	0.04	2.06	11.11
41	3-Hexanone	C_6_H_12_O	84.17521	0.44	0.03	2.20	10.20
42	4,11,13,15-Tetrahydroridentin B	C_15_H_24_O_4_	268.0904	0.48	0.04	2.02	1.36
43	3-Methylhistidine	C_7_H_11_N_3_O_2_	169.0778	0.26	0.04	2.26	0.86
44	3,7,11,15-Tetramethyl-6,10,14-hexadecatrien-1-ol	C_20_H_36_O	276.8187	2.48	0.04	1.78	0.73

## Data Availability

The data generated from the study is clearly presented and discussed in the manuscript.
